# Characterization of Equine Infectious Anemia Virus Integration in the Horse Genome

**DOI:** 10.3390/v7062769

**Published:** 2015-06-19

**Authors:** Qiang Liu, Xue-Feng Wang, Jian Ma, Xi-Jun He, Xiao-Jun Wang, Jian-Hua Zhou

**Affiliations:** State Key Laboratory of Veterinary Biotechnology, Harbin Veterinary Research Institute of Chinese Academy of Agricultural Sciences, Harbin 150001, China; E-Mails: liuqiang_yyy@163.com (Q.L.); xuefengwang1982@126.com (X.-F.W.); jma@hvri.ac.cn (J.M.); hexijun@caas.cn (X.-J.H.)

**Keywords:** equine infectious anemia virus, integration sites, chromosomes, RefSeq genes, repetitive elements

## Abstract

Human immunodeficiency virus (HIV)-1 has a unique integration profile in the human genome relative to murine and avian retroviruses. Equine infectious anemia virus (EIAV) is another well-studied lentivirus that can also be used as a promising retro-transfection vector, but its integration into its native host has not been characterized. In this study, we mapped 477 integration sites of the EIAV strain EIAV_FDDV13_ in fetal equine dermal (FED) cells during *in vitro* infection. Published integration sites of EIAV and HIV-1 in the human genome were also analyzed as references. Our results demonstrated that EIAV_FDDV13_ tended to integrate into genes and AT-rich regions, and it avoided integrating into transcription start sites (TSS), which is consistent with EIAV and HIV-1 integration in the human genome. Notably, the integration of EIAV_FDDV13_ favored long interspersed elements (LINEs) and DNA transposons in the horse genome, whereas the integration of HIV-1 favored short interspersed elements (SINEs) in the human genome. The chromosomal environment near LINEs or DNA transposons potentially influences viral transcription and may be related to the unique EIAV latency states in equids. The data on EIAV integration in its natural host will facilitate studies on lentiviral infection and lentivirus-based therapeutic vectors.

## 1. Introduction

Integration, *i.e*., the incorporation of viral cDNA into a host cell genome, is a crucial step in the retrovirus life cycle that is mediated by preintegration complexes (PICs), which are composed of viral integrase, cellular proteins and other viral proteins [1,2,3]. As a result, retroviruses, in the form of proviruses, become an integral part of the host’s chromosomes, persist for the duration of the host’s lifetime and establish latent infections in the host cell. The irreversible integration of retroviruses makes them promising therapeutic vectors for human genetic diseases. Retrovirus-based vectors have been successfully used in human gene therapy to correct primary immunodeficiency and genetic deficiency [4,5,6,7,8]. However, adverse events, such as disruption of genes and activation of the proto-oncogenes, have raised awareness of the safety implications of retroviral-based vectors used in clinical gene therapy [9,10,11,12]. Not all retroviral-based vectors have the same genotoxic potential. For example, gammaretroviruses tend to cause disruption of genes more than lentiviruses [13,14,15,16,17,18]. Therefore, studies on the selection of integration sites for retroviruses in host genomes, especially for gene therapy vectors, have particular significance.

The complete human genome is available online, and data on the integration sites of some important retroviruses have been surveyed, which have shown that selection of the target site is not random and that different retroviruses have different preferences in the human genome. For example, the lentiviruses in the human genome, e.g., human immunodeficiency virus type 1 (HIV-1) [19], simian immunodeficiency virus (SIV) [20], and feline immunodeficiency virus (FIV) [21], are prone to insert into transcription units, particularly in actively transcribed host genes. Furthermore, murine leukemia virus (MLV) tends to integrate near transcription start sites (TSSs) or CpG islands [22]. In contrast, target site selection for avian sarcoma-leukosis virus (ASLV) has been shown to be random, and ASLV displays only a slight preference for transcription units during its infection of human-derived cell lines [23,24].

Equine infectious anemia virus (EIAV) is an important etiological agent to equids and has served as an animal model for HIV-1/AIDS research [25,26]. Most infected horses eventually become asymptomatic carriers of the virus after several months of the initial infection with a low plasma viral load and viral replication occurring in tissues rich in mononuclear cells [27]. However, the viral replication and clinical symptoms can be reactivated by natural or experimental suppression of the horse’s immunity [28,29]. It is known that the chromosomal environment around the integration sites influences the replication and gene expression of the integrated provirus [30,31,32]. What, therefore, are the integration site characteristics of EIAV in the genome, and particularly in the horse genome? Hacker *et al*. [33] published a study in 2006 on EIAV integration in the human genome in HEK293T cells using a vesicular stomatitis virus G protein (VSV-G)-pseudotyped, EIAV-based transfection vector that was prepared using a three-plasmid system. In comparison with an HIV-1-based vector control, EIAV exhibited a preference similar to that of HIV-1 integration in the human genome. Both of these lentiviral vectors tended to insert within genes, especially actively transcribed genes, and they also favored AT-rich regions [33,34]. However, to the best of our knowledge, there is no published study on the integration of EIAV or other retroviruses in the chromosomes of equids.

EIAV_FDDV13_ is an EIAV strain that is adapted to cultivated equine and donkey fibroblasts (fetal dermal cells), which are among the few EIAV-permissive equine cells and are widely used for EIAV infection and replication *in vitro*. This virus’s genomic, virological and immunological characteristics have been extensively studied [35,36,37]. With the availability of the complete horse genome sequence [38,39], it is possible to map EIAV integration sites in horse chromosomes. In this study, fetal equine dermal (FED) cells were infected with EIAV_FDDV13_, and 477 EIAV integration sites were cloned, sequenced, and mapped to the horse genome. In addition, because the human genome database has been updated and additional information on EIAV integration in the human genome also needs to be analyzed, the published data on the integration sites of EIAV and HIV-1 in the human genome were also downloaded and processed using our analysis pipeline in this study for reference to our data. This study, which evaluates the features of EIAV integration in the horse genome, provides additional knowledge on lentiviral integration.

## 2. Materials and Methods

### 2.1. Cell Preparation and Infection

FED cells were cultured and infected with EIAV as described previously for fetal donkey dermal (FDD) cells [40]. Briefly, FED cells were plated in 75 cm^2^ flasks (Corning, Corning, NY, USA) for 2 days until they reached approximately 90% confluency. EIAV_FDDV13_ was inoculated at a multiplicity of infection (MOI) of 10 for 1 h to allow the adsorption and infection of the virus. The cells were then washed twice with phosphate buffered saline (PBS) and once with fresh α-minimal essential medium (α-MEM) (Life Technologies, Carlsbad, CA, USA) containing 10% fetal bovine serum (FBS) (Sigma, St Louis, MO, USA), and then they were re-cultured in the same medium at 37 °C under 5% CO_2_. It is known that lentiviral cDNA enters the nucleus within 24 h post-infection, and viral particles are produced and released within 48 h post-infection [41,42]. To maximize provirus formation but minimize clonal expansion during cell growth after integration [43], the FED cells were harvested 24 h post-infection. The harvested cells were stored at −80 °C until further use.

### 2.2. Cloning and Sequencing of Integration Sites

Cellular genomic DNA (gDNA) was extracted using a QIAamp DNA mini kit (Qiagen, Hilden, Germany), according to the manufacturer’s instructions. A library of integration site junctions containing 371 bp of the 5′ end of the integrated EIAV long terminal repeat (LTR) and the up-stream horse gDNA was created by ligation-mediated PCR (LM-PCR) performed as previously described by Ciuffi *et al.* [43,44]. Briefly, to remove unintegrated (circular and linear forms) viral DNA, 2 mg of gDNA was electrophoresed on a 0.6% agarose gel on ice. DNA bands longer than 8.3 kb were extracted from the gel, purified using an E.Z.N.A. TM Ploy Gel DNA Extraction Kit (Omega, Stamford, CT, USA) and digested overnight with the restriction endonucleases *Ssp* I or *Dra* I to fragment the long gDNA. The digested DNA fragments were blunt-ligated to a double-stranded adapter, which was prepared by annealing from Link-1 and Link-2.

The ligation products were amplified by a two-step PCR. The first PCR amplification was performed with an adaptor-specific primer, APF1, and a viral specific primer, EIAV514. The PCR reaction conditions were as follows: 5 min of pre-denaturation at 98 °C, 7 cycles of denaturation at 98 °C for 25 s and an extension step at 72 °C for 3 min, followed by 32 cycles of denaturation at 98 °C for 25 s and extension at 68 °C for 3 min. A final extension was performed at 72 °C for 10 min. The PCR products were then diluted using TE buffer (Invitrogen, Carlsbad, CA, USA) at a ratio of 1:50 (*v*/*v*), and 1 μL of diluted amplified DNA was used as the template for the secondary PCR reaction. The reaction was performed with an adaptor-specific primer, APF2, and a viral-specific primer, EIAV359. The conditions of the reaction were identical to those of the first-round PCR except that the number of amplification cycles was reduced (20 cycles). The PCR products were visualized using 2% agarose gel electrophoresis, and the diffuse bands longer than 371 bp were extracted and purified using a gel extraction kit (Qiagen, Germany) and then cloned using the TOPO TA cloning kit (Invitrogen, Carlsbad, CA, USA). All clones were cultured and sequenced.

### 2.3. Sequences Analysis and Mapping of Integration Sites in the Host Genome

For reference to EIAV integration in the horse genome, 562 human genome sequences flanking the 3′ LTR for the retrovirus, which included 146 integration sites for an HIV-1 transfection vector (DQ498202 to DQ498347) and 416 sites for an EIAV vector (DQ498348 to DQ498763), were downloaded from GenBank and processed using our analysis pipeline in this study (Table 1). These vectors were produced using a three-plasmid lentiviral vector system, which included an EIAV-based transduction vector and vectors encoding the EIAV gag/pol and the VSV-G envelope. These vectors were then co-transfected into 293T cells as described in a previous report [33]. The number of sequences in GenBank was less than that published by Hacker *et al*. [33], which had been analyzed and mapped to the human genome (University of California, Santa Cruz (UCSC), assembled in May 2004). In this study, the human genome database, which has been recently updated, was used to analyze and map these sequences (UCSC, assembled in December 2013).

**Table 1:** Datasets for the integration sites used in this study.

Group	Virus or Vector	Cell Type	Number of Integration Sites	Accession Numbers (GenBank)	Source of Reference
HIV-1	HIV-1 vector	HEK 293T ^a^	146 ^b^ (162 ^c^)	DQ498202–DQ498347	[33]
EIAV	EIAV vector	HEK 293T	416 (458)	DQ498348–DQ498763	[33]
EIAV*-Ssp* I	EIAV_FDDV13_^d^	FED ^e^	287	KO454413–KO454699	This study
EIAV*-Dra* I	EIAV_FDDV13_	FED	190	KO454223–KO454412	This study

^a^: Human embryonic kidney 293T cells; ^b^: Actual number of sequences downloaded from the NCBI database provided by Hacker *et al.* [33]; ^c^: Number of sequences reported by Hacker *et al*. [33]; ^d^: EIAV fetal equine dermal cell-adapted strain; ^e^: Fetal equine dermal cell.

The Blast-like Alignment Tool (BLAT) program (Available online: http://genome.ucsc.edu) was used to analyze the sequences, which were aligned and mapped to the horse genome (UCSC, assembled in September 2007) (Available online: http://genome.ucsc.edu/). A site was deemed to be an integration site for EIAV if it complied with the following criteria: (1) must be located between the adaptor sequence and the EIAV LTR; (2) must have at least 95% homology to the horse genome sequence and should be a single horse genetic locus; (3) the junction must consist of a horse genomic sequence and 371 bp of the 5′ terminal of the EIAV LTR, within which “TGTGGG” must be used as the initial sequence based on the sequence of EIAV_FDDV13_; and (4) must have a minimum length of 20 bp, which was the lowest limit recognized by the BLAT program. The sequences that were used for reference were aligned and mapped to the recently updated human genome (UCSC, assembled in December 2013) (Available online: http://genome.ucsc.edu/).

The BioMart program (Ensemble Genes 79, Available online: http://asia.ensembl.org) was used to determine whether the integration sites were located in coding genes of the September 2007 horse genome draft. Additional information regarding transcription initiation and termination sites of the coding genes was also obtained from the BioMart program. The reference mRNA sequence (RefSeq mRNA) of the September 2007 horse genome draft was downloaded from the UCSC genome annotation database (Available online: http://www.genome.ucse.edu). The base frequency around the integration sites was determined using the WebLOGO program (Available online: http://weblogo.berkeley.edu/). The repetitive elements around the integration sites were determined by the RepeatMasker analysis of the September 2007 horse genome draft (Available online: http://www.repeatmasker.org/).

### 2.4. Statistical Analysis

To determine integration site selection bias *in vitro*, a set of 10,000 random integration sites was generated using the Microsoft Excel “RAND()” function by choosing random numbers between 1 and 2,367,055,132, which represents the total length of the 31 autosomal chromosomes plus the X sex chromosome in the horse genome (UCSC, assembled in September 2007) as previously described for the generation of a random dataset [33]. All statistical analyses were performed using the statistics analysis system (SAS) 9.2. The one-sided Fisher exact or chi-squared test was used to determine whether the differences in chromosome distribution were significant and to determine the integration frequencies into gene coding regions and repetitive elements. *p* ≤ 0.05 and *p* ≤ 0.01 were considered significant or very significant, respectively, for all tests performed.

### 2.5. Nucleotide Sequence Accession Numbers

The integration site sequences in this study were uploaded to GenBank. The accession numbers range from KO454223 to KO454699.

## 3. Results

### 3.1. Distribution of EIAV Provirus Integration Sites in Horse Chromosomes

To determine the horse genomic sequences flanking the EIAV provirus integration sites, genomic (g)DNA from FED cells infected with EIAV_FDDV13_ was isolated, digested with restriction endonucleases, and ligated to a DNA adaptor. Integration sites were amplified using the 5′ primer that bound the adaptor and the 3′ primer that bound to the provirus long terminal repeat (LTR), and then the PCR products were cloned and sequenced. To reduce the integration bias introduced by usage of restriction endonucleases in chromosomes, two different restriction endonucleases, *Ssp* I and *Dra* I, were used to single-digest the horse gDNA. Based on the criteria for authentic integration sites described in the Materials and Methods, we obtained a total of 477 horse genomic sequences flanking the EIAV integration sites, which included 287 sequences obtained from the *Ssp* I-digested (*Ssp* I group) and 190 sequences from the *Dra* I-digested (*Dra* I group) horse gDNA. These sequences were analyzed and mapped to the horse genome (UCSC, assembled in September 2007) (Table 1). In addition, as references for the analysis of EIAV integration in the horse genome, 562 human genomic sequences flanking the lentivirus integration sites, including 146 sequences for HIV-1 and 416 sequences for EIAV, were downloaded and mapped to an updated human genomic database (UCSC, assembled in December 2013) (Table 1).

**Figure 1:**
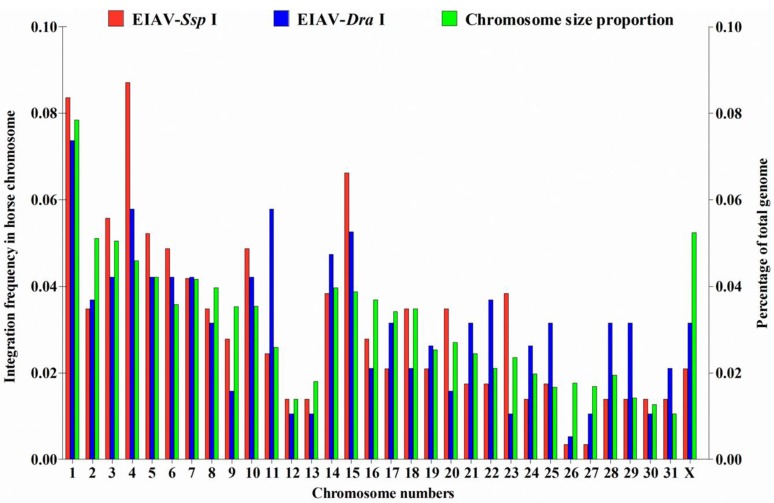
Chromosome distribution of EIAV_FDDV13_ integration sites in horse chromosomes. Chromosomal DNA of FED cells infected with EIAV_FDDV13_ was digested with the restriction endonuclease *Ssp* I or *Dra* I. Genomic fragments with EIAV integration sites were subcloned after being amplified by LM-PCR. The proportion of integration sites in horse chromosomes digested with *Ssp* I (287 sites) or *Dra* I (190 sites) is indicated as a percentage, and the percentage of each corresponding chromosome size was calculated based on the length of the entire horse genome. The chi-squared test was used.

Mapping of both the *Ssp* I and *Dra* I groups showed that integration events occurred in all 31 autosomal chromosomes plus the X sex chromosome in the horse genome. No significant differences were observed between the *Ssp* I and *Dra* I groups with respect to the integration frequency in each chromosomes (*p* > 0.05) (Figure 1). Furthermore, although variations in insertion events and chromosome sizes appeared in the EIAV-integrated horse genome, no evidence of significant differences in the distribution of integration sites was found among chromosomes (*p* > 0.05) (Figure 1). Furthermore, the human genomic sequences flanking HIV-1 and EIAV integration sites in human chromosomes were also analyzed to determine their chromosomal distribution. Integration occurred in all 22 autosomal chromosomes plus the X sex chromosome in the human genome (except the eighteenth chromosome for EIAV). The integration frequencies of HIV-1 in chromosomes 3, 4 and 6 were higher than those of EIAV (*p* < 0.05). In contrast, the integration frequencies of EIAV in chromosomes 15–17 and 19 were higher than those of HIV-1 (*p* < 0.05) (Figure S1).

### 3.2. Specificity of the 40 bp Region around the Integration Sites

**Figure 2:**
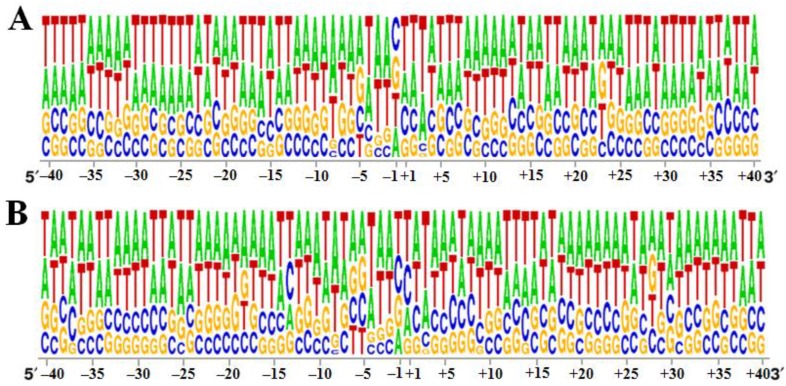
Base frequency within 40 bp around the integration site of EIAV_FDDV13_ in the horse genome. (**A**) *Ssp* I-digested horse genome; (**B**) *Dra* I-digested horse genome. The diagrams were generated using the WebLOGO program (Available online: http://weblogo.berkeley.edu/). The relative lengths of the nucleotides on the Y-axis represent the frequency of each base.

After retroviral integration into host chromosomes, the primary sequence is found to play a minor role in the selection of the target sites, but a weak palindromic sequence is often observed at the insertion sites of retroviruses, including HIV-1, MLV and ASLV [45,46]. In this study, the 40 bp sequence around the integration sites was analyzed. We found that as for other lentiviruses, AT-rich regions were characteristic of the target site selection (Figure 2A,B). In addition, in both the *Ssp* I and *Dra* I groups, a palindromic sequence centered around the insertion site (centered between the site −1 of the palindromic sequence for the *Ssp* I group and +1 for the *Dra* I group) was observed in the horse genome (Figure 2A,B). However, these characteristics were not observed in the corresponding regions of EIAV and HIV in the human genome. In contrast, a palindromic structure centered at +3 between the −1 and +6 position from the insertion sites was observed at the integration sites of HIV-1 and EIAV in human chromosomes (Figure S2A,B). Our study found that EIAV integration sites, particularly in the *Dra* I group, also had a weak palindromic sequence centered around +3 in the horse chromosomes (Figure 2A,B).

### 3.3. EIAV_FDDV13_ Provirus Tended to Integrate into Host RefSeq Genes

A previous study on lentiviral integration examining HIV-1, FIV and SIV integration into the human genome found that integrations more frequently occurred in regions between the transcriptional start and stop sites of genes from the Reference Sequence (RefSeq) database [19,20,21]. In the present study, we asked whether this pattern occurred in the integration of the EIAV provirus into the horse genome. Our results showed that 53.0% (152/287) of integrations in the *Ssp* I group and 55.3% (105/190) in the *Dra* I group occurred in RefSeq genes. The integration rate was not notably different between these two groups (*p* > 0.05) (Table 2), but it was significantly higher than those from a set of 10,000 computer-generated random insertion sites, of which only 33.2% occurred in RefSeq genes (*p* < 0.01) (Table 2). We also analyzed the frequency of integration of the EIAV and HIV-1 in RefSeq genes from human chromosomes using the updated genome database (UCSC, assembled in December 2013). In total, 77.4% (113/146) of HIV-1 and 75.2% (313/416) of EIAV integrations occurred in RefSeq genes, which is generally consistent with the 72% (116/162) for HIV-1 and 68% (311/458) for EIAV *(P* > 0.05) previously reported by Hacker *et al*. [33] in 2006 (Table 2). The integration frequencies for both EIAV and HIV-1 in the human chromosomes were significantly higher than that of EIAV in horse chromosomes observed in this study (*p* < 0.01).

**Table 2:** Comparison between groups for the integration sites within genes.

	Random	HIV-1 vector	EIAV vector		EIAV-*Ssp* I group				EIAV-*Dra* I group				
	No. (%)	No. (%)	No. (%)	*P2* ^d^	No. (%)	*P1* ^c^	*P2* ^d^	*P3* ^e^	No. (%)	*P1* ^c^	*P2* ^d^	*P3* ^e^	P4 ^f^
Total	10000 (100.0)	146 (100.0)	416 (100.0)	NA	287 (100.0)	NA	NA	NA	190 (100.0)	NA	NA	NA	NA
In RefSeq genes ^a^	3315 (33.2)	113 (77.4)	313 (75.2)	NS	152 (53.0)	**	**	**	105 (55.3)	**	**	**	NS
Sense ^b^	ND (ND)	65 (57.5)	149 (47.6)	NS	79 (52.0)	NA	NS	NS	63 (60.0)	NA	NS	*	NS

RefSeq genes represent reference sequence genes; ^a^: The percentage of the provirus integrated in RefSeq genes was calculated and indicated in brackets; ^b^: The percentage of sense-direction insertion in the genome was calculated among RefSeq genes in which the viral genome was integrated; ^c^*: P1* shows a comparison with the random group using the chi-squared test; ^d^*: P2* shows a comparison with the HIV-1 vector group using the chi-squared test; ^e^: *P3* shows a comparison with the EIAV vector group using the chi-squared test; ^f^: *P4* shows a comparison with the *Ssp* I group using the chi-squared test; NA indicates not applicable; ND indicates not done. NS indicates not significant; * indicates significant, *i.e*., *p* < 0.05; ** indicates very significant, *i.e*., *p* < 0.01.

### 3.4. EIAV_FDDV13_ Integration Targeted Low Gene Density Regions and Avoided the TSS

Our results presented above suggest that EIAV_FDDV13_ has a preference for integration into RefSeq genes, *i.e*., the transcription units. Therefore, we further investigated some other characteristics of the genome that may relate to retrovirus integration. These characteristics include (a) the insertion orientation relative to RefSeq genes; (b) the gene density within a 2 Mb region around the integration site; (c) the integration frequency near the TSS.

**Figure 3:**
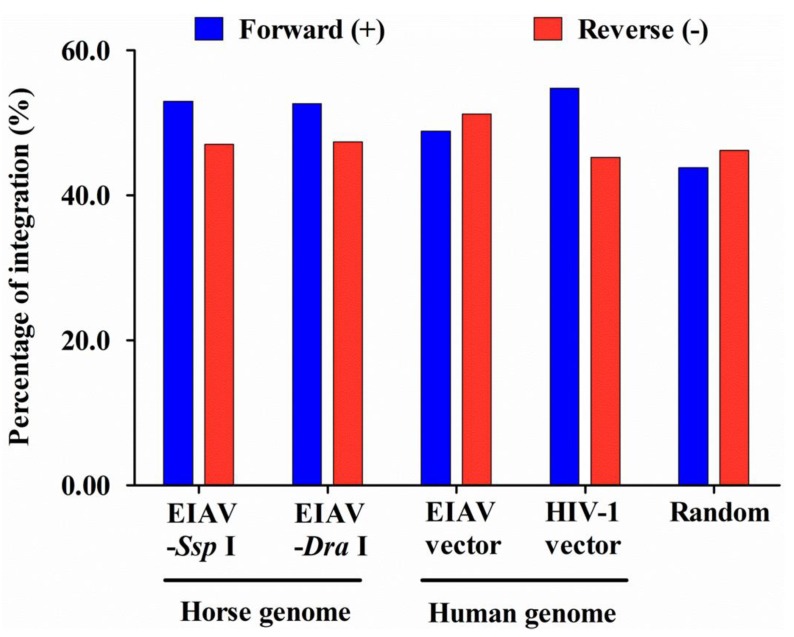
Orientation of the integration sites relative to the TSS. The percentages were calculated according to the total number of integration sites in each type of sample. The chi-squared test was used.

**Figure 4:**
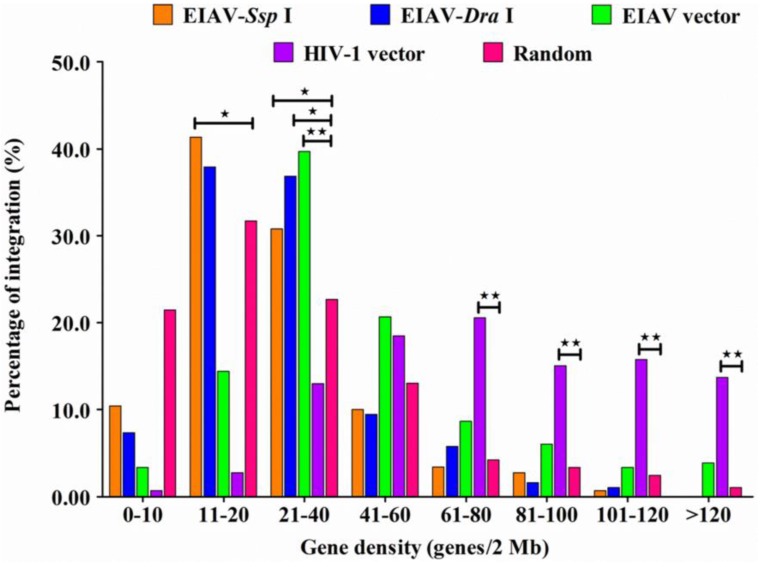
Gene density within a 2 Mb window centered on the insertion site was determined and classified into eight groups (0–10, 11–20, 21–40, 41–60, 61–80, 81–100, 101–120, >120). The percentage of integration within each group was calculated. The number of integration sites analyzed in the *Ssp* I and *Dra* I group was based on the horse genome; and the HIV-1 and EIAV vector group was based on the human genome. The chi-squared test was used. * indicates significant, *i.e*., *p* < 0.05; ** indicates very significant, *i.e*., *p* < 0.01.

**Figure 5:**
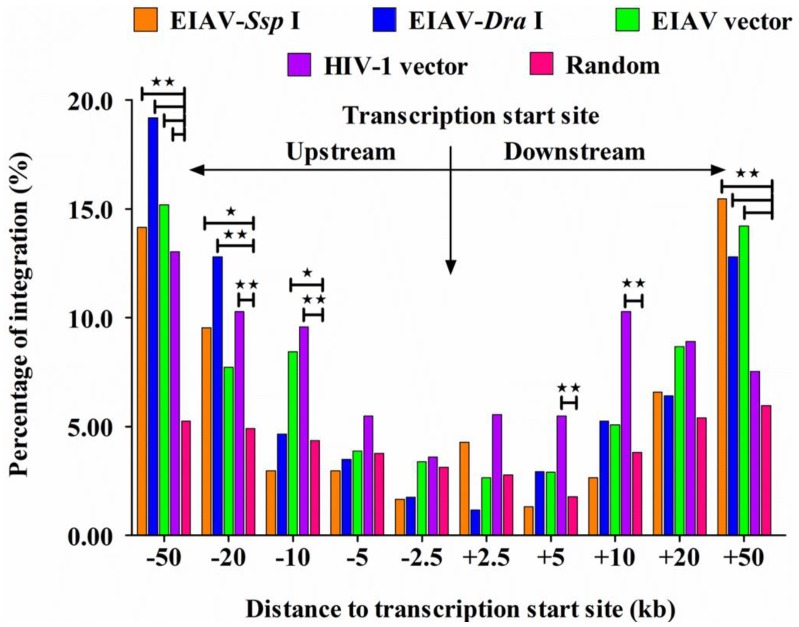
Frequencies of integration at different nucleotide windows up- and downstream of TSS. Five symmetric variable-size windows near the TSS in the host genome were indicated, and their corresponding frequencies were calculated (0–2.5, 2.5–5.0, 5.0–10, 10–20, and 20–50 kb). The number of integration sites analyzed in the *Ssp* I and *Dra* I group was based on the horse genome; and the HIV-1 and EIAV vector group was based on the human genome. The chi-squared test was used. * indicates significant, *i.e*., *p* < 0.05; ** indicates very significant, *i.e*., *p* < 0.01.

Our results showed that no differences existed in the insertion orientation either for the total integration sites (Figure 3) or for the sites integrated into RefSeq genes in both horse and human genome (Table 2). In addition, the frequencies of insertion orientation were similar between the *Ssp* I and *Dra* I groups of the EIAV integrated into the horse genome and the EIAV and HIV-1 integrated into the human genome (*p* > 0.05). Furthermore, analytic data revealed that EIAV_FDDV13_ appeared to have a preference for integration into regions with low (11–20 RefSeq/2 Mb for *Ssp* I groups) or moderate gene density (21–40 RefSeq/2 Mb for *Ssp* I and *Dra* I groups) in the horse chromosomes (*p* < 0.05), which was similar to the mean value of gene density in the horse chromosomes (the mean value was 29.0 RefSeq/2 Mb). In contrast, our analytic data showed that integration events of EIAV tended to occur in regions of moderate gene density (21–40 RefSeq/2 Mb) in the human chromosomes (39.3 RefSeq/2 Mb) (*p* < 0.05). However, HIV-1 tended to occur in regions of high gene density (>61 RefSeq/2 Mb) in the human chromosomes (*p* < 0.01) (Figure 4), which was consistent with previously published results [33]. Our data also revealed that EIAV_FDDV13_ integration generally avoided the TSS in the horse chromosomes, similarly to the results obtained on the transfection of EIAV and HIV-1 vectors into human cells (Figure 5).

### 3.5. Integration Frequency of EIAV_FDDV13_ in Repetitive Sequences

A previous study on HIV-1 integration sites in repetitive elements indicated that this virus favors *Alu* repeats of the short interspersed nuclear elements (SINEs) family, and no bias was seen in insertion in the long interspersed nuclear elements (LINEs) [19,46]. In this study, we investigated whether EIAV integration in horse repetitive elements exhibited preferences. Repetitive sequences consist of approximately half of the horse genome (49.5%), and contain four major classes of transposable elements (TEs): LINE retrotransposons, SINE retrotransposons, DNA transposons and LTR transposons [39]. The relative proportions of the various repetitive sequences in the horse or human genome were obtained from a previous report [39,47] and are listed in Table 3 and Table S2, respectively.

Regarding the EIAV_FDDV13_ integration sites that occurred in repetitive elements, 69 of 120 sites (57.5%) from the *Ssp* I group and 38 of 69 sites (55.1%) from the *Dra* I group were found to occur in LINEs. Compared to the 37.9% of integration within LINEs among the matched random insertion sites occurred in repetitive elements, EIAV had a significant preference for integration within LINEs in the horse genome (*p* < 0.01) (Table 3). Similar analytic results were obtained when these integration data were compared with a set of sites of matched random control (MRC) generated based on the methods as described by Mitchell *et al*. [24] and Berry *et al*. [48]. Conversely, the frequency of EIAV_FDDV13_ integration in SINEs of horse chromosomes was 12.5% (15 of 120 sites) for the *Ssp* I group and 13.0% (9 of 69 sites) for the *Dra* I group. Considering the portions of integration within LINEs and SINEs among the matched random insertion sites occurred in repetitive elements and the insertion sites in each of the elements (37.9% and 13.8%, respectively), EIAV appeared to prefer to integrate in LINEs compared to SINEs in the horse genome (*p* < 0.01) (Table 3). In contrast, the EIAV and HIV-1 had similar frequenciy of integration into both LINEs (37.5% for EIAV and 25.9% for HIV-1) and SINEs (36.3% for EIAV and 49.4% for HIV-1) in the human genome (Table S2). Compared with the proportions of integration within LINEs or SINEs among the matched random insertion sites occurred in repetitive elements (40.2% or 29.7%, respectively), the HIV-1 favored SINEs to LINEs in the human genome (*p* < 0.01) (Table S2).

**Table 3:** Correlation between repetitive elements and EIAV integration sites in the horse genome.

	Random control	EIAV-*Ssp* I		EIAV-*Dra* I		
	No. (%) ^a^	No. (%)	*P1* ^c^	No. (%)	*P1* ^c^	*P2* ^d^
Total ^b^	4876	120		69		
LINEs	1848 (37.9)	69 (57.5)	**	38 (55.1)	**	NS
SINEs	675 (13.8)	15 (12.5)	NS	9 (13.0)	NS	NS
DNA transposons	322 (6.6)	21 (17.5)	**	15 (21.7)	**	NS
LTR transposons	658 (13.5)	11 (9.2)	NS	6 (8.7)	NS	NS

^a^: Percentages of repetitive elements in the host genome were based on the total numbers of integration sites inserted into repetitive elements in the random control; ^b^: The number of integration sites inserted into the repetitive elements was calculated based on the horse genome; ^c^: *P1* displays a comparison of the frequency of corresponding repetitive elements in the random control using the chi-squared test; ^d^: *P2* presents a comparison with *Ssp* I group integration using the chi-squared test. NS indicates not significant; ND indicates not determined; * indicates significant, *i.e*., *p* < 0.05; ** indicates very significant, *i.e*., *p* < 0.01.

In addition, the *Ssp* I group had 17.5% (21/120) and the *Dra* I group had 21.7% (15/69) of integration sites in repetitive sequences located in DNA transposons of the horse genome, percentages that were significantly higher than the proportion of integration within DNA transposons among the matched random insertion sites occurred in repetitive elements in the horse genome (*p* < 0.01) (Table 3). Similarly, 20 of 160 insertions (12.5%) of EIAV were identified in DNA transposons of human repetitive sequences, a level that was significantly higher than the proportion of integration within this type of element among the matched random insertion sites occurred in repetitive elements in the human genome (*p* < 0.01) (Table S2). However, HIV-1 did not display any preference for DNA transposons in the human genome (*p* > 0.05) (Table S2). The differences in the integration preferences within other repetitive elements were not significant.

## 4. Discussion

In this study, we examined the integration characteristics of an FED cell-adapted EIAV strain, EIAV_FDDV13_, in the horse genome. Our results demonstrated that during infection of FED cells, one of the most frequently used types of host cells for *in vitro* EIAV studies, the integration sites of EIAV_FDDV13_ provirus were not randomly distributed but instead were prone to fall within RefSeq genes. Overall, these data implied that EIAV_FDDV13_ shares several similarities in integration site selection with other lentiviruses, such as HIV-1 [19], SIV [20], and FIV [21] but obviously differs from MLV and ASLV in the human genome [22,23]. Notably, our results showed that EIAV_FDDV13_ preferred to integrate into LINEs compared to SINEs and other repetitive elements in the horse genome, while HIV-1 tended to insert within SINEs in the human genome. More interestingly, EIAV favored integration within DNA transposable elements in both the horse and human genomes, but HIV-1 did not display this preference in the human genome. Therefore, this study showed some unique integration features of EIAV_FDDV13_ integration in horse genomes, and it also revealed similarities of EIAV integration site selection in the horse and human genomes, which improves our understanding of lentiviral integration and latency.

The usage of restriction enzymes to digest host gDNA during viral integration site cloning could have introduced biases, as this method tends to isolate integration sites near the restriction sites. However, the observation of integration in *in vitro* experimental controls suggested that the methods used for isolation and analysis of integration sites did not detectably bias the conclusions [19]. Additionally, various cloning and analytic strategies for examining HIV-1 integration sites in the human genome, including the fragmentation of gDNA with restriction enzymes or physical approaches [49], amplification with inverse PCR (I-PCR) [50] or LM-PCR [19,30] and high-throughput sequencing [49,51], obtained similar conclusions on the integration characteristics in different types of human cells [48,52,53,54]. Similarly, consistent integration features were observed for MLV in the human genome using different restriction enzymes and PCR methods [22,54,55,56]. Therefore, these techniques all could effectively clone retroviral integration sites without causing significant bias. In the present study, two different restriction endonucleases, *Ssp* I and *Dra* I, were applied for LM-PCR of EIAV insertion sites in FED cells, which further increased the unbiased nature of the cloning of gDNA fragments that integrated with EIAV provirus.

EIAV_FDDV13_ integration clearly tended to occur in RefSeq genes. Similar patterns of integration targeting have been reported for other lentiviruses [19,20,21]. However, the frequency of integration of EIAV_FDDV13_ in these gene-containing regions of the horse genome was significantly lower than that of the integration of HIV-1 and SIV (72% for HIV-1 and 74% for SIV) [20] or EIAV and HIV-1 in the human genome as analyzed in this study (77.4% and 75.2%, respectively). It is logical to presume that this difference may be partially attributed to the incomplete annotation of RefSeq genes in horse chromosomes. Although the horse genome shares extensive conserved synteny with the human genome, the total size is smaller (2.5–2.7 Gb for horses compared with 2.9 Gb for humans), and only 26,740 RefSeq genes have been identified in horse chromosomes (ENSEMBL Genes 79), which is significantly fewer than the 59,492 genes identified in human chromosomes (ENSEMBL Genes 79). In addition, different types of cells from the same host or cells from different species of hosts have variant replication rates and activation states, which might alter interactions between viral pre-integration complex (PIC) and host cell proteins and thereafter influence EIAV integration in RefSeq genes. These factors may partially explain our observation of a relatively low integration frequency of EIAV in RefSeq genes in the horse genome. Certainly, the specific structures and functions of viral proteins in each type of virus also affect insertion rates in RefSeq genes.

The results from this study and others revealed some specific integration features among different species of retroviruses. It is known that integration site selection in the human genome is largely determined by viral integrases and cellular factors that bind to and interact with integrases [57,58]. Indeed, integrases from all lentivirus members share highly conserved structural features. Lens epithelium-derived growth factor (LEDGF), which is conserved in natural hosts of lentiviruses, interacts with lentiviral integrases by forming a complex and mediates viral integration in the host chromosomes [59,60]. In addition, viral proteins other than integrase have also been shown to affect integration site selection in HIV-1. Studies using HIV-1/MLV chimeric viruses indicated that HIV-1 capsid proteins have implicated a role in influencing target site preference for integration [57,61]. In addition, recent studies have demonstrated that the bromo- and extra-terminal domain (BET) protein, which mediates gammaretrovirus integration near the transcription initiation site, is a major cellular factor that covalently binds MLV integrase [62,63,64]. Our data on some unique integration characteristics of EIAV_FDDV13_ from this study provide additional evidence for the specific integration preference of the *Retroviridae* genus of lentivirus.

Our analysis revealed that the EIAV provirus is more prone to integrate in LINEs than in SINEs in the horse genome. Concurrently, consistent with some publications, HIV-1 prefers inserting in SINEs (especially *Alus*) in the human genome compared to LINEs ([19] and this study). There are two possible explanations for these differences: The first explanation is that host proteins in cells of different origins involved in retroviral integration are in different cell types and have different activities. Because the integration of the EIAV strain in horse cells favored LINEs but the HIV in human cell line 293 preferred SINEs, this possibility is at least partially responsible for this difference. The second explanation involves the different copy numbers and ratios of LINEs to SINEs between the horse and human genome. These two types of elements consist of 19.7% and 7.0% of the horse genome and 20% and 13% of the human genome, respectively. The lower percentage of SINEs identified in the horse genome could occur because (1) the horse genome contains fewer SINE elements; or (2) the horse genome has been incompletely annotated, which is less likely because we compared the proportion of LINEs and SINEs from clones of the same genome and it is unlikely that only SINEs were largely unidentified in the genomic database. For other lentiviruses, the chromosomal environment around the integration sites influences the replication and gene expression of the integrated provirus [30,31,32,50]. LINE elements are significantly enriched in GC- and gene-poor regions [65]. Genes with high expression usually do not occur near LINEs, and the presence of LINEs will reduce the transcriptional activity of genes, possibly due to the presence of a strong polyA signal within LINEs, which can destroy transcription elongation [66]. Most horses infected with EIAV will eventually become asymptomatic carriers [25,26]. The integration site preference, including in LINEs and DNA transposons, is presumed to be one possible driving force that contributes to this infection behavior.

DNA transposon sequences, which are able to move from one genomic location to another by a cut-and-paste mechanism (transposition), make up approximately 3% of the host genome [47]. Currently, DNA transposons, in particular *Sleeping Beauty* (SB), a member of the Tc1/mariner family of DNA transposons, are most widely used as a gene therapy tool for human genetic diseases [67]. Studies on integration site selection found that the integration of SB was nonrandom in the host chromosome. This transposon strongly favored integration into non-coding regions, including heterochromatin and microsatellite repeats [68]. Using an HIV-1-based vector transducing GFP in Jurkat cells, Lewinski *et al*. [30] reported that inducible expression (similar to the latent state) of GFP was more frequently identified in (1) very highly expressed host genes; (2) gene deserts; and (3) heterochromatin. These results suggest that the chromosomal environment surrounding the DNA transposons’ insertion sites influences the activity of the transposons. In this study, EIAV tended to insert into DNA transposons in the horse and human genome. It is logical to infer that the activation of EIAV proviruses integrated within DNA transposons could be suppressed by the chromosomal environment surrounding the DNA transposon insertion sites, which may be one of the reason that EIAV tend to be silenced in long-term infected horse.

The EIAV vector, similarly to other lentiviral vectors such as HIV-1, has certain advantages as a therapeutic gene transfer vector. It is important to determine what integration is like for the EIAV vector in horse genome, *i.e*., if the same result that was observed in human cells would be seen in equid cells. We recently completed the construction of a three-plasmid, VSV-G pseudotyped EIAV transfection system, which will be used together with a three-plasmid HIV-1 transfection system, to compare the integration of EIAV vector in the horse genome to that of the human genome.

## Supplementary Files

Supplementary File 1
