# Supplementary material for: Characterization of Equine Infectious Anemia Virus Integration in the Horse Genome
Source: Viruses. 2015 Jun 19;7(6):3241–60. doi: 10.3390/v7062769 (PMC4488736; doi:10.3390/v7062769)
Supplement: Supplementary File 1 [file viruses-07-02769-s001.pdf]

Supplementary Information

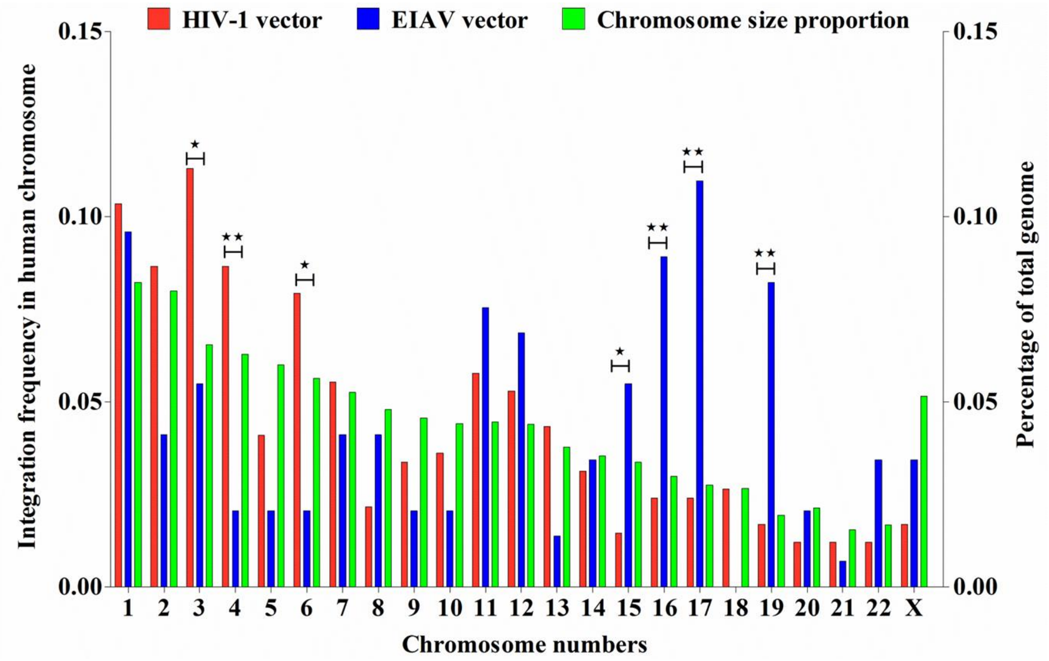

Figure S1. Chromosome distribution of viral integration sites of HIV-1 and EIAV transfection vectors in human chromosomes (146 and 416 sites, respectively). The integration sites were cited from GenBank and re-analyzed using the updated human genomic database (UCSC, assembled in December 2013). The proportion of integration sites in human chromosomes is indicated as a percentage, which is compared with the percentage of each corresponding chromosome size based on the length of the whole human genome. The chi-squared test was used.

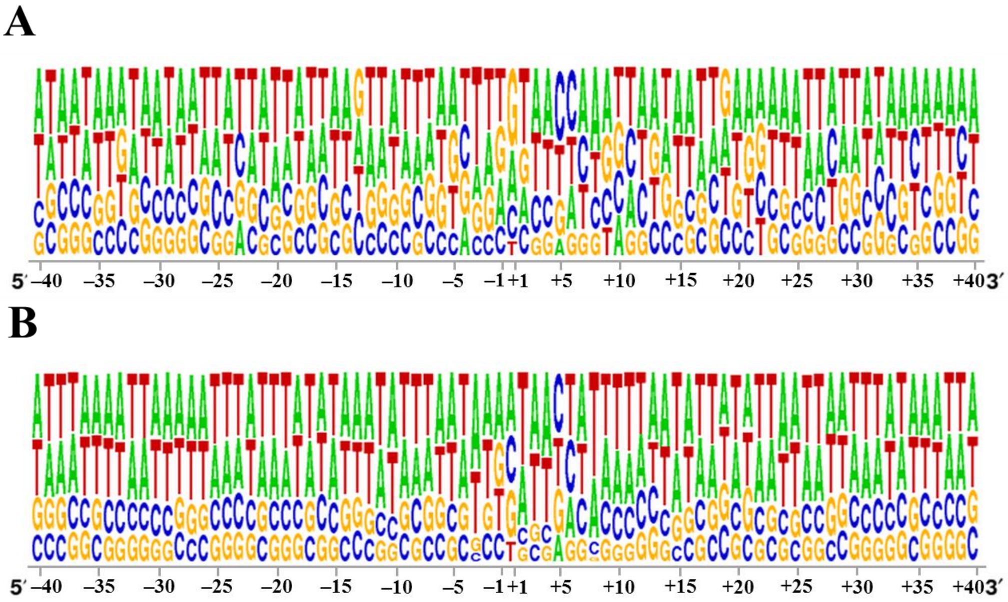

Figure S2. Base frequency within 40 bp around the integration sites of HIV-1 and EIAV transfection vectors in the human chromosome. (A) Base frequency of HIV-1 vector integration sites. (B) Base frequency of EIAV vector integration sites. The diagrams were generated using the WebLOGO program (available online: http://weblogo.berkeley.edu/). The relative length of four nucleotides on the Y-axis represents the frequency of each base.

Table S1. PCR primer sequences.

Name	Primer sequence (From 5′ to 3′)
AD-L	CTAATACGAGTCACTATAGCGCTCGAGCGGCCGCCGGGGAGGT
AD-S	PO_4-ACCTCCCC-NH_2
APF1	GGATCCTAATACGAGTCACTATAGCGC
EIAV514 [a]	CTTTTGAGACCCTTGTACCGTCACCTT
APF2	CTATAGCGCTCGAGCGGC
EIAV359 [b]	GGTTCAGCAGGCAGGGTCT

a/b: Primer sequences were designed according to the EIAV complete genome (GenBank: AF327877).

Table S2. Correlation between repeat elements and integration sites in the human genome.

	Random control	HIV vector		EIAV vector		
	No. (%) [a]	No. (%)	*P1* [c]	No. (%)	*P1* [c]	*P2* [d]
Total [b]	4647	81		160		
LINEs	1869 (40.2)	21 (25.9)	**	60 (37.5)	NS	NS
SINEs	1292 (27.8)	40 (49.4)	**	58 (36.3)	*	0.05
DNA transposons	310 (6.7)	4 (4.9)	NS	20 (12.5)	**	NS
LTR transposons	824 (17.7)	10 (12.3)	NS	19 (11.9)	NS	NS

a: Percentages of repetitive elements in the host genome were based on the total numbers of integration sites inserted into repetitive elements in the random control; b: The number of integration sites inserted into the repetitive elements was calculated based on the human genome; c: *P1* displays a comparison of the frequency of corresponding repetitive elements in the random control using the chi-squared test; d: *P2* presents a comparison with HIV-1 vector group integration using the chi-squared test. NS indicates not significant; * indicates significant, *i.e.*, $p < 0.05$;
** indicates very significant, *i.e.*, $p < 0.01$.
